# Operon information improves gene expression estimation for cDNA microarrays

**DOI:** 10.1186/1471-2164-7-87

**Published:** 2006-04-21

**Authors:** Guanghua Xiao, Betsy Martinez-Vaz, Wei Pan, Arkady B Khodursky

**Affiliations:** 1 Division of Biostatistics, School of Public Health, University of Minnesota, A460 Mayo Building, Minneapolis, MN 55455-0378, USA; 2 Department of Biochemistry, Molecular Biology and Biophysics, University of Minnesota, Saint Paul, MN, 55108, USA

## Abstract

**Background:**

In prokaryotic genomes, genes are organized in operons, and the genes within an operon tend to have similar levels of expression. Because of co-transcription of genes within an operon, borrowing information from other genes within the same operon can improve the estimation of relative transcript levels; the estimation of relative levels of transcript abundances is one of the most challenging tasks in experimental genomics due to the high noise level in microarray data. Therefore, techniques that can improve such estimations, and moreover are based on sound biological premises, are expected to benefit the field of microarray data analysis

**Results:**

In this paper, we propose a hierarchical Bayesian model, which relies on borrowing information from other genes within the same operon, to improve the estimation of gene expression levels and, hence, the detection of differentially expressed genes. The simulation studies and the analysis of experiential data demonstrated that the proposed method outperformed other techniques that are routinely used to estimate transcript levels and detect differentially expressed genes, including the sample mean and SAM t statistics. The improvement became more significant as the noise level in microarray data increases.

**Conclusion:**

By borrowing information about transcriptional activity of genes within classified operons, we improved the estimation of gene expression levels and the detection of differentially expressed genes.

## Background

Genome-wide monitoring of transcription by means of DNA microarrays is used to infer transcriptional and regulatory networks in living organisms. In most of microarray experiments, transcript levels of thousands of genes are measured with a relatively small number of replications, so the estimates of true expression levels from microarray data may be poor, mostly due to a small sample size. To address this problem, several statistical methods have been proposed to borrow information from other genes to improve detection of the differentially expressed ones [1-9]. The main idea is to borrow information from other genes to estimate either the distributions of genes's expression levels or the distribution of error terms. The underlying assumption is that it should be possible to improve the estimates of expression levels of genes by borrowing information about transcriptional activity across the sets of genes that are biologically, or physically, related. In some cases, the expression levels may significantly vary across the genes, then borrowing information from unrelated genes may not improve, or even worsen, the estimates of gene expression levels [10]. However, if, based on biological knowledge, we can expect that some genes are more likely to express at similar levels (i.e. co-express), then we can improve the inference by using information about the activity of those genes.

An operon [11] is a set of linearly juxtaposed genes transcribed as a single mRNA; operons are commonly found in prokaryotic genomes such as *Escherichia coli*. Transcription of operons of *E. coli *has been examined, and operons have been predicted in many studies [12-19], which provides background information about the *E. coli *regulatory network. The genes within the same operon usually have similar expression levels, hence show some local structure in expression profiles [20], and this fact has been successfully used in operon prediction [21,22].

Based on the existing information about the structure of operons, we propose a hierarchical Bayesian model which improves gene expression estimation by borrowing information from genes within the same operon. Most existing methods for detecting differently expressed genes [1-9] borrow information from other genes in the whole genome, while our proposed method only borrows information from other genes within the same operon, which has sound biological basis. Wren *et al *[23] have proposed a simulated annealing approach to adjust gene expression data by using existing microarray measurements obtained on the same organism, which effectively reduced the noise and made it possible to compare different microarray experiments. But their method relies on reference microarray experiments that cover the dynamic range of transcript abundances for most of the genes, which may be difficult to select or unavailable. Instead of using existing microarray measurements, we use existing information about the operons' structure to reduce the noise in microarray data.

A more accurate estimation of transcript abundances of individual genes will improve our ability to evaluate transcriptional activity on a genome-wide scale, and hence facilitate the exploration of gene regulatory networks. Our proposed method provides a better way to estimate relative transcript levels, which is critical for distinguishing differentially expressed (DE) genes from equally expressed (EE) genes. Herein, we refer to the logarithm of a ratio of the fluorescent intensities of the test and control samples as the observed gene expression level. The genes with estimated expression levels significantly different from zero are identified as DE genes, otherwise as EE genes.

Using more than 200 microarray experiments, we obtained the evidence of co-transcription of genes within *E. coli *operons on a genome-wide scale. We applied the proposed method to three simulated and one experimentally obtained data sets. The simulation studies and the real data application demonstrated that the proposed method performed better than the sample mean and the SAM t statistics in estimating the gene expression levels as well as in detecting differentially expressed genes. The improvement became more significant as the noise level in microarray data increased.

## Results

### Simulation study

We carried out three simulations, with similar settings and the noise level gradually increasing from simulation 1 to 3. In simulations, we assumed that the genes within an operon are co-transcribed. Since the true expression levels in a simulated data set were known, we could calculate the mean squared errors of the estimated expression levels (summarized in Table 1). Without incorporating the information from operons, the sample mean of the observed expression levels would be a natural estimator of a gene's expression level. The mean squared errors of the two estimates, the posterior mean from the proposed model and the sample mean, are shown in Table 1 for comparison. It can be easily seen that the incorporation of operon information leads to a better estimate, with a smaller mean squared error, of expression levels. When the noise level increased, the improvement from incorporating operon information also increased.

**Figure 1:**
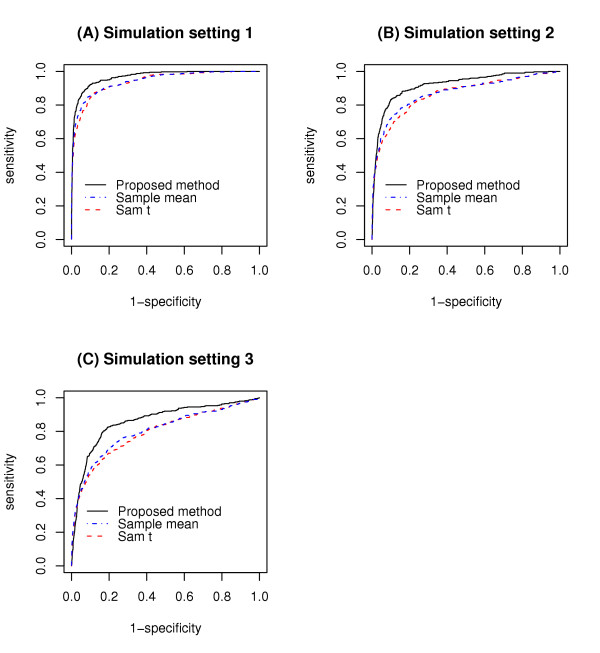
**ROC curves of simulation settings**. The Figure (A), (B), and (C) are the ROC for simulations 1,2 and 3, respectively. It shows that the sample mean and the SAM t statistic have similar performance in detecting DE genes, and our hierarchical model outperformed both of them.

To evaluate the performance of the proposed method in detecting DE genes, the estimated expression level of each gene was used to rank the genes, and the highly ranked genes were identified as DE genes. Since the identities of DE genes in the simulation studies were known, we compared the performances of the proposed method, sample mean, and SAM t statistics in detecting DE genes using receiver operating-characteristic (ROC) curves (Figure 1). In a ROC curve, the *sensitivity *is plotted against 1 – *specificity*. The sensitivity is denned as a fraction of true DE genes being correctly detected and the specificity is a fraction of the true EE genes being correctly identified. The ROC curves in Figure 1 demonstrate that the performance of the sample mean and of the SAM t statistic were very close, and our hierarchical model, incorporating operon information, outperformed both of them. The difference in the performance became greater as the noise level increased. For example, as the specificity equals to 0.8, the sensitivities of the methods using the sample mean or SAM t were about 0.91, 0.80, and 0.68 for simulations 1,2, and 3, respectively, while the sensitivities of the proposed method were 0.95, 0.89 and 0.83, respectively.

### Application to E. coli data

**Figure 2:**
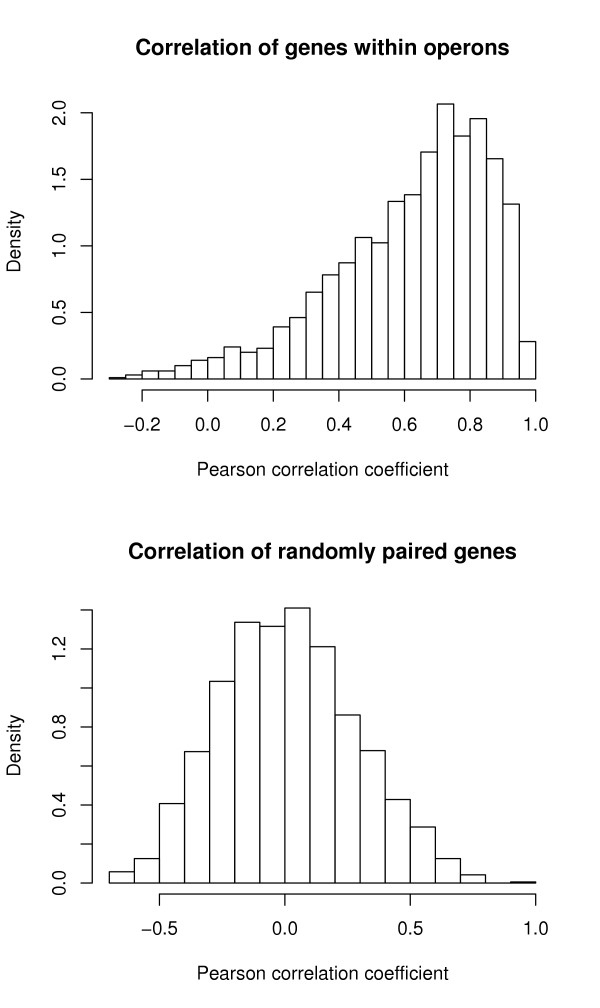
**Histogram of the correlation coefficients**. Histogram of pairwise correlation coefficients for (A)the genes within operons and (B) random gene pairs. The correlations are calculated across experimental conditions. The correlation of genes organized in operons is much higher than that of random genes, strongly indicating the co-expression of genes within operons.

To verify the assumption that the genes organized in operons are co-expressed, we pooled together data from 217 microarray experiments, obtained in 53 conditions [24]. The distribution of pairwise correlations between expression profiles of genes in operons was greatly skewed towards positive values, with the mean correlation of 0.62 (Figure 2A). Unlike the profiles of genes organized in operons, expression profiles of randomly picked pairs of genes were not correlated; the corresponding distribution of correlation coefficients was almost symmetric around 0, with the mean correlation of 0.012 (Figure 2B). This result demonstrated the similarity of transcriptional activity of genes within operons and served as a motivation for borrowing information from other genes within the same operon.

**Figure 3:**
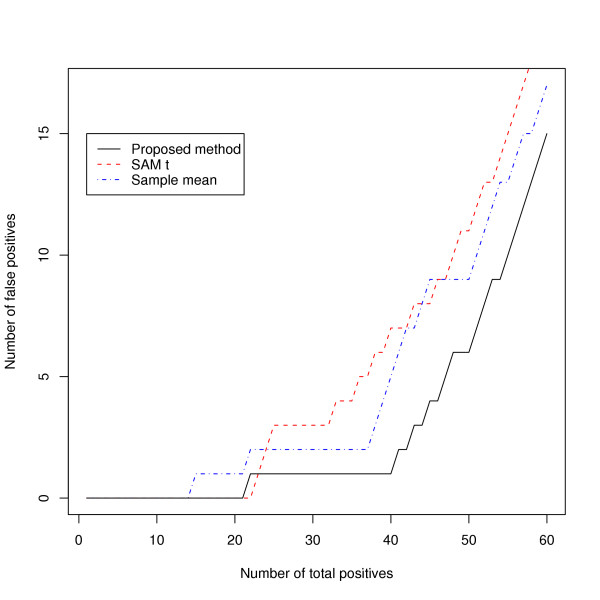
**Number of false positives vs. Number of total positives**. For the *E. coli *motility data, the genes are ranked by using the proposed method, SAM t statistic and sample mean. The number of false positives is plotted against the number of total positives for each ranking criterion. It shows that ranking genes by proposed method has less false positives than ranking genes by SAM t or sample mean.

The proposed method [see Additional file 1] was used to analyze differential transcriptional activity in an *E. coli *mutant lacking the *flhDC *gene, a master regulator of transcription of genes whose products mediate bacterial motility and chemotaxis [see Additional file 2]. The genes were ranked by their estimated expression levels, i.e. their posterior means of *μ_*i *_*obtained from the proposed model. For the sake of comparison, the sample mean and SAM t statistics were also used to rank the genes [see Additional file 3]. Using the functional annotation from Macnab [25] as a standard, we obtained the number of false positives at different cut-off levels (total positives). The comparison revealed that ranking genes by the proposed method produced fewer false positives than the ranking based on the SAM t or sample mean statistics (Figure 3).

**Figure 4:**
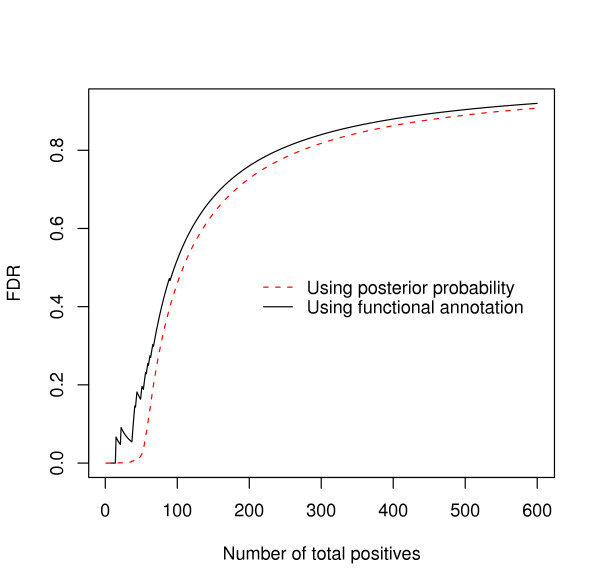
**FDR estimation**. Estimate FDR by using posterior probability and the functional annotation from Macnab *et al*. The solid line is for the FDR estimate using existing functional annotation while the dashed line for using posterior probability. It indicates that estimate the FDR by using posterior probability yields reasonable result.

To find a reasonable cutoff value for differently expressed genes, we calculated the false discovery rate (FDR) [26,27]based on the posterior probability [7]. In this experiment, we also estimated the FDR by using the functional annotation from Macnab [25] as a reference. Comparison of the estimated False Discovery Rates revealed that the estimated FDR from the posterior probability was a little lower than that derived from the annotation, which could be due to the partial incompleteness of the reference (Fig. 4). Overall, the estimated FDR from the posterior probability is close to the FDR using the reference list of genes, indicating that our method for estimating the FDR is adequate. We set the cutoff for the FDR to be 0.01, which identified the top 44 genes as DE genes. At such a cutoff, the estimated number of false negatives is 14 and the estimated false negative rate is about 0.003 (see the "Methods" section for details). The top 44 genes are listed in Table 2. Note that, in Table 2, the gene expression level is on the log scale and the FDR corresponds to a specific number of DE genes and not to each individual gene itself. According to Macnab's classification [25], 41 genes out of the 44 were expected to be differentially expressed in the *flhDC- *dependent manner, whereas the lists of 44 genes identified by using SAM t and sample mean contained only 36 and 38 expected genes, respectively.

We examined some genes and operons in more detail, to demonstrate the advantages of borrowing information from within an operon. For example, an operon *argT-hisJQMP *contains 5 genes (argT, hisJ, hisM, hisP, hisQ) and is not expected to be differentially expressed under the examined experimental condition, according to our biological knowledge [25]. But from the microarray data, the mean expression level (an average log ratio) of the gene *argT was *-2.73, which ranked 15th among all the expression levels in the *E. coli *genome. This "high expression" of the *argT *could possibly be caused by random noise in microarray measurements and/or in biological samples, since the mean expression levels of other genes within the same operon were close to 0 (those for genes hisP, hisM, hisQ, hisJ were 0.00, -0.05, 0.03, and 0.05, respectively). However, accounting for expression levels of other genes within the same operon lowered the estimate of the expression level (posterior mean of *μ_*i *_*of the *argT to *-0.02, rank of 1456, indicating that this gene was not differentially expressed. Analysis of another operon, *fliDST*, illustrates a complimentary case. Transcription of the *fliDST operon *(containing 3 genes, *fliD, fliS, fliT*) is known to be controlled by the FlhDC [25], and thus, under the experimental condition, differential expression of genes in that operon would be expected. While the expression level of the *fliT*, estimated by the sample mean at -0.90, ranked only 65th, the estimated expression level of the gene after borrowing information from two other genes in the operon was -3.25, which ranked 19th.

**Figure 5:**
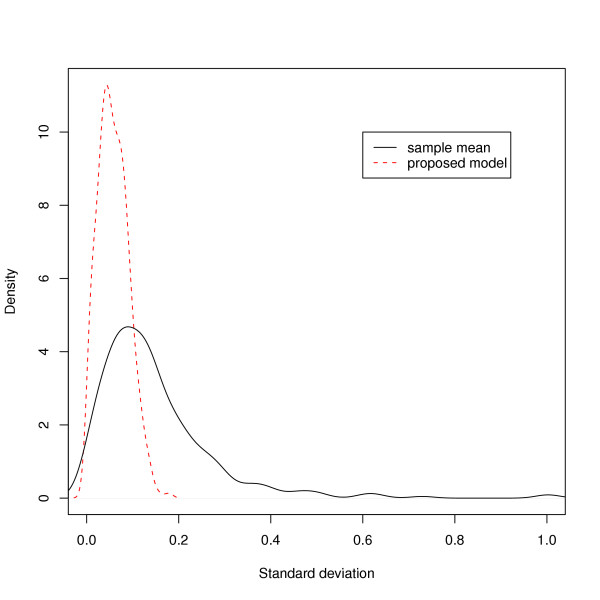
**Within-operon standard deviation of the estimated gene expressions**. Distribution of the standard deviation of the expression estimates of the genes from the same operon. The solid line is for the sample mean method while the dashed line for our proposed Bayesian model. Comparing to the sample mean method, our proposed method yields smaller within-operon standard deviations of the gene expressions.

In general, through borrowing information, our Bayesian method worked in a way giving more consistent estimates of the expression levels for the genes of the same operon. For example, compared with using the sample mean to estimate expression levels, the Bayesian method tended to yield smaller standard deviations of the expression estimates for within-operon genes (see Figure 5).

## Discussion

In this paper, we proposed and applied a hierarchical Bayesian model, to estimate relative gene expression levels and detect differentially expressed genes by borrowing expression information within operons. The performance of the proposed method was compared with that of the sample mean and SAM t statistics. Through the simulation studies, we showed that the proposed method outperformed the sample mean and the SAM t statistics in estimating gene expression levels and detecting DE genes. The proposed method was used to analyze differential expression in an *E. coli *mutant with a defect in transcription of motility/chemotaxis genes, giving results more consistent with the existing biological knowledge than those obtained by using the other statistics.

A major advantage of the proposed approach is in borrowing expression information from other genes within the same operon. The approach is developed within a statistically sound Bayesian model and it offers necessary flexibility with respect to the amount of information that needs to be borrowed from other genes. By borrowing information we can obtain stabilized estimates of expression levels from rather noisy microarray data. As a result, the estimates of transcript levels within the same operon become more similar to each other, more so than without borrowing information; this is consistent with a biological fact that genes within the same operon are transcribed as a single mRNA molecule. With the proposed method, the estimated expression levels of genes in "differentially expressed" operons are consistently high, and more importantly, transcript abundances of genes in "equally expressed" operons are stabilized towards zero. In the experimentally obtained microarray data, the within operon variation was smaller than the variation among the replicate data points for the same genes, indicating that the expression levels of the genes within an operon were very similar.

In our model, the ratio of parameters *τ*^2 ^and  determines how much information comes from the observed expression of a gene and how much comes from the average expression of an operon, when estimating the expression level of an individual gene within an operon. A smaller *τ*, as compared to , puts more weight on the average expression of an operon. Here we assumed the same *τ *value for all operons, implying that all operons had similar within-the-operon variability. However, this assumption might not be realistic. In some operons and physiological conditions, the genes might express very similarly, but in others, especially under the control of internal promoters, transcription of individual genes may be more heterogeneous. In the future, we will investigate the effect of an operon-specific *τ*. Operonal organization of genes is common in prokaryotes and also present in some eukaryotic organisms [28-30], and the proposed method can be extended to biological systems where the operonal structure is unknown. Many biological studies have demonstrated that co-expressed genes tend to cluster on the chromosome. Although the nature of this phenomenon is not quite understood, a positional clustering of co-expressed genes can be found in many eukaryotes including yeast [31,32], worm [33], fly [34,35], mouse [36], and human [37-39]. These findings indicate that the genes are likely to co-transcribe with their chromosomal neighbors. In those cases, instead of borrowing information from genes in the same operon, we can borrow information from gene neighbors on the chromosome. Another extension of our method would involve incorporation of gene annotation information into the analysis of expression data. The approach would be very similar to the one described in this paper: based on biological knowledge, the genes belonging to the same functional group are more likely to be co-expressed, so we can use a hierarchical model to borrow information from and for the genes within the same functional group to improve the estimates of gene expression levels.

## Conclusion

The information about operon structure leads to a better estimation of gene expression levels. Using simulated and experimental data sets, we have demonstrated that the proposed method performs better than the sample mean and the SAM t statistics in estimating the relative levels of transcript abundances and detecting differentially expressed genes.

## Methods

### RegulonDB database and E. coli. microarray data

RegulonDB [40] is a database containing information about known operons in *E. coli*. According to the RegulonDB annotations, 1486 genes (about one third of all genes predicted in the *E. coli *genome) are organized in 600 operons.

The *E. coli *data set contains results of 217 microarrays collected in 53 different experimental conditions. The fluorescent intensities of the test and control samples were measured, and the average log ratio of the intensities for each gene under the same condition was used here to represent an observed gene expression level under that condition [24].

An *E. coli *motility expression data set ([41] series accession number: GPL2101) was obtained in a direct pair-wise comparison between a knock-out mutant of the *flhDC*, a master regulator of the motility/chemotaxis regulon [25], and its isogenic wild type strain. Total RNA samples of a mutant *E. coli *(test samples) and an isogenic wild type *E. coli *(control samples) were labeled with red (Cy5) and green (Cy3) fluorophors. The intensities from the red and the green channels were normalized by the lowess method [42]. There were 4281 genes (G = 4281) with four replicates for each gene (n = 4). Let *Y*_*i*,*j *_be defined as the log ratio of the intensities between the test and control samples for gene *i *on array *j; *that is,



### Hierarchical models

We propose a hierarchical Bayesian model,



where, *Y*_*i*,*j *_is the log ratio of gene *i *in replicate *j, μ_*i *_*and *σ*_*i *_are the true expression level and the standard deviation, respectively. In our method, the posterior mean of *μ_*i *_*is used as the estimated expression level of gene *i*, while the sample mean, ., is referred to as the observed expression level.

As prior knowledge, we assume that if several genes belong to the same operon, in accordance with the RegulonDB annotation, then their expression levels are from a normal distribution, with the mean *λ*_*p *_and the variance *τ*^2^. Specifically,



where *O*_*p *_denotes operon *p*. *λ*_*p *_represents the expression level of the operon *p*, which is the average of the mean expression levels of all genes within the operon *p*. *τ*^2 ^is the within operon variation, and is assumed to be the same across all operons. A non-informative prior is assigned to *λ*_*p*_, that is *Pr*(*λ*_*p*_) α 1, to reflect the lack of prior information,  and *τ*^2 ^have vague priors, which are inverse Gamma distributions with the shape and rate parameters equal to 0.01 and 0.01 respectively [43]. If gene *i *is not in any operon, then



so the posterior mean of *μ_*i *_*is just the the sample mean ; if gene *i *is in operon *p*, then the conditional distribution of *μ_*i *_*can be derived as:



where



Equation (3) shows that, when borrowing information from the other genes within the same operon, the estimated expression level of the gene *i *becomes the weighted average of the observed expression level of gene *i *and the expression level of operon *p*, given that gene *i *belongs to operon *p*. The weights are inversely proportional to the variances. In this model, a key concept is to shrink the observed expression level , towards *λ*_*p*_, the expression level of an operon, based on the knowledge of the operon structure. The degree of shrinkage is determined by the variability of . and *λ*_*p*_. Without incorporating operon information, the estimated expression level would be close to the observed expression level,  In the hierarchical model, *λ*_*p *_represents the expression level of operon *p*, and



where, *m*_*p *_is the number of genes in operon *p*, and *i *∈ *O *_*p *_denotes that gene *t *is in operon *p*. Although the posterior distribution was not available in a closed form, we could derive a closed form of the full conditional distribution, and used Markov chain Monte Carlo (MCMC) to simulate the parameters from the posterior distribution. With this closed form expression, the model could be easily coded in R [see Additional file 1] for MCMC simulation using Gibbs sampling [44]. The expression level of gene *i *is estimated by the posterior mean of *μ_*i*_*, and the genes are ranked by the absolute values of the posterior means of the *μ_*i*_'*s. Genes with high rankings were designated as differentially expressed (DE) genes.

### SAM t statistic

To evaluate the performance of the proposed method in estimating gene expression levels and identifying DE genes, we compared the proposed method to the sample mean and SAM t statistics [1,3]. Because of its good performance, the SAM t statistic [1,3] is widely used to rank genes and detect DE genes [45]. We denote the SAM t statistic for the gene *i *as *Z*_*i*_, then



where  and *S*_*i *_are the sample mean and sample standard deviation for the gene *i*, and *So *is the 90 *th *percentile of *S*_*i*_'s.

### Simulation settings

We conducted three simulation studies to assess the usefulness of our method. The operon structure of the *E. coli *genome from the RegulonDB database [40] was used in simulation studies. We randomly chose 100 operons (involving about 340 genes) and assumed that genes from those operons were differentially expressed DE genes. Then we randomly picked a subset of non-operon genes to be DE genes and adjusted the total number of DE genes to 400. Let *μ_*i *_*be the expression level of gene *i*, for *i = *1, 2,..., 4821. For DE genes, *μ_*i*_'*s were simulated from an equal mixture of *N*(1, 0.25^2^) and *N(–*1,0.25^2^) distributions, and genes within the same operon were from the same component of the mixture distribution. For EE genes, *μ*_*i *_~ *N*(0, 0.25^2^). We simulated 4 replicates from a normal distribution for each gene, *Y*_*ij *_~ *N*(μ_*i*_,), where *Y*_*i*,*j *_was the log ratio of transcript abundances for the gene *i *on the array *j*. To provide increasing noise levels for simulations 1, 2 and 3, the σ_*i*_'s were simulated from the *uniform*(0.25, 0.75), *uniform(*0.5,1.0), and *uniform(*0.75,1.25), respectively.

### Estimation of false positives and false negatives

Using the posterior distributions, we can evaluate the FDR for specific number of DE genes. Using *Pr*(|*μ*_*i*_| > *δ'*|*Y*_*i,j*_) to estimate the probability of gene *i *to be a DE gene, we can estimate the number of false positives for a cut off value *k *[7]:



Here, the genes are ranked based on the estimated mean expression level *μ_*i*_*. In this study, we set δ = 1,

which corresponding to the commonly used 2-fold cutoff.

The false discovery rate (FDR) for the cut off *k *can be derived as:



Similarly, the number of false negatives for the cut off *k *can be calculated as:



### Algorithm for Gibbs sampler

The algorithm is implemented below:

Set initial values:



FOR t FROM 1 TO T, draws random samples:



#### END FOR

where *V*_*i *_is the sample variance of gene *i*, and *V*_0 _is the median of *V*_*i*_'s. *n*_*p *_is the number of genes in operon *p*. *T *is the total number of iteration. To diminish the effect of the initial values, we discard the results from the early iterations *(t ≤ T*_*B*_, where *T*_*B *_is the burn in time). The posterior mean of *μ*_*i*_, of gene *i *is calculated by:



In our proposed method, the expression level of gene *i *is estimated by . In the real data example, *T*_*B *_and *T *are 500 and 2000, respectively.

## Authors' contributions

GX initiated the study, implemented the methods and conducted data analysis. WP participated in development of the methods and co-wrote the paper. BMV generated the *E. coli *motility data. ABK generated the *E. coli *data set containing 217 microarrays, supervised the project and co-wrote the paper. All authors contributed to the writing, read and approved the final manuscript.

## Supplementary Material

Additional File 1 Operon.r – The R code used in the study. Click here for file

Additional File 2 Motility.txt – E. coli motility data set. Click here for file

Additional File 3 Result.txt – The result for E. coli motility data, including posterior mean of proposed method, sample mean and SAM t statistics Click here for file

## Figures and Tables

**Table 1:** Mean squared errors for different methods

	Proposed model	Sample mean	Difference
Setting 1	0.062	0.068	8.6%
Setting 2	0.129	0.146	12.8%
Setting 3	0.222	0.255	15.1%

**Table 2:** Top 44 genes and their estimated relative transcription levels

Name	B number	Operon	Verified	Estimated expression	FDR
fliC	B1923		+	-4.71	0.000
flgB	B1073	flgBCDEFGHIJK	+	-3.90	0.000
flgE	B1076	flgBCDEFGHIJK	+	-3.82	0.000
flgL	B1083		+	-3.81	0.000
flgF	B1077	flgBCDEFGHIJK	+	-3.81	0.000
flgD	B1075	flgBCDEFGHIJK	+	-3.81	0.000
flgK	B1082	flgBCDEFGHIJK	+	-3.76	0.000
flgI	B1080	flgBCDEFGHIJK	+	-3.76	0.000
flgJ	B1081	flgBCDEFGHIJK	+	-3.75	0.000
flgH	B1079	flgBCDEFGHIJK	+	-3.74	0.000
flgC	B1074	flgBCDEFGHIJK	+	-3.73	0.000
flgG	B1078	flgBCDEFGHIJK	+	-3.71	0.000
fliA	B1922	fliAZY	+	-3.44	0.000
fliY	B1920	fliAZY	+	-3.35	0.000
fliZ	B1921	fliAZY	+	-3.32	0.000
fliD	B1924	fliDST	+	-3.30	0.000
fliS	B1925	fliDST	+	-3.25	0.000
fliT	B1926	fliDST	+	-3.25	0.000
tap	B1885		+	-3.22	0.000
tar	B1886		+	-2.97	0.000
tsr	B4355		+	-2.64	0.000
fadL	B2344		-	-2.37	0.001
fliH	B1940	fliFGHIJK	+	-2.03	0.001
fliG	B1939	fliFGHIJK	+	-2.01	0.001
fliF	B1938	fliFGHIJK	+	-1.99	0.001
fliK	B1943	fliFGHIJK	+	-1.99	0.001
fliJ	B1942	fliFGHIJK	+	-1.92	0.000
flgA	B1072	flgAMN	+	-1.91	0.001
fliI	B1941	fliFGHIJK	+	-1.89	0.001
flgM	B1071	flgAMN	+	-1.87	0.001
flgN	B1070	flgAMN	+	-1.81	0.001
flxA	B1566		+	-1.70	0.002
aer	B3072		+	-1.66	0.002
cheZ	B1881		+	-1.63	0.002
cheR	B1884		+	-1.60	0.003
fliM	B1945	fliMNOPQR	+	-1.55	0.003
cheY	B1882		+	-1.51	0.005
fliL	B1944	fliMNOPQR	+	-1.50	0.005
fliN	B1946	fliMNOPQR	+	-1.49	0.005
ycgR	B1194		-	-1.44	0.007
cheW	B1887	motAB-cheAW	+	-1.42	0.007
cheA	B1888	motAB-cheAW	+	-1.39	0.007
b1904	B1904		-	-1.38	0.009
mot A	B1890	motAB-cheAW	+	-1.32	0.009

Note: the estimated gene expression levels is log scale.
